# Supplementary material for: Reward learning and working memory: Effects of massed versus spaced training and post-learning delay period
Source: Mem Cognit. 2021 Sep 14;50(2):312–24. doi: 10.3758/s13421-021-01233-7 (PMC8821056; doi:10.3758/s13421-021-01233-7)
Supplement: Supplementary file 1 — (DOCX 38 kb) [file 13421_2021_1233_MOESM1_ESM.docx]

**Supplementary Results**

*Choices across massed and spaced conditions*

We also collected choices between massed- and spaced-trained stimuli, comparing reward- versus reward-associated stimuli and loss- versus loss-associated stimuli. In the No-Delay group, for reward-associated stimuli, we found that participants preferred massed-trained stimuli over the spaced-trained stimuli (massed versus spaced: 64.2% CI [56.4 71.9]; t_(26)_ = 3.767, p < 0.001), consistent with the strength of working memory-supported values for massed-trained stimuli at no delay. For loss-associated stimuli, we found no difference in preference (51.5 % CI [39.0 58.0]; t_(26)_ = 0.334, p = 0.74; TOST p = 0.007). Reflecting the shift in relative preference across the delay, in the Delay group we found that participants showed no preference for massed- or spaced-trained stimuli (reward-associated massed versus spaced: 52.5% CI [43.8 61.1]; t_(26)_ = 0.588, p = 0.56; TOST p = 0.007; loss-associated: 49.7% CI [40.1 59.3]; t_(26)_ = -0.066, p = 0.95; TOST p = 0.004).

Comparing performance in these mixed choices across the No-Delay and Delay groups, we found that for reward-associated stimuli, preferences for massed- versus spaced-trained stimuli were significantly lower in the Delay versus No-Delay group (reward difference: -11.7% CI [-23.0 -0.4]; t_(52)_ = -2.079; p = 0.043; loss difference, -1.9% CI [-15.0 11.3]; t_(52)_ = -0.28; p = 0.779; TOST p = 0.011). This relative decrease in value for massed and increase in value for spaced reward-associated stimuli after rest parallels the changes in performance from learning to test.
